# Does the National Health Insurance Scheme provide financial protection to households in Ghana?

**DOI:** 10.1186/s12913-015-0996-8

**Published:** 2015-08-15

**Authors:** Anthony Kusi, Kristian Schultz Hansen, Felix A Asante, Ulrika Enemark

**Affiliations:** Section for Health Promotion and Health Services Research, Department of Public Health, Aarhus University, Vennelyst Boulevard 6, 8000 Århus C, Denmark; Department of Global Health and Development, Faculty of Public Health and Policy, London School of Hygiene and Tropical Medicine, 15-17 Tavistock Place, WC1H 9SH London, UK; Institute of Statistical, Social and Economic Research (ISSER), University of Ghana, P.O. Box LG 74, Legon, Accra, Ghana

## Abstract

**Background:**

Excessive healthcare payments can impede access to health services and also disrupt the welfare of households with no financial protection. Health insurance is expected to offer financial protection against health shocks. Ghana began the implementation of its National Health Insurance Scheme (NHIS) in 2004. The NHIS is aimed at removing the financial barrier to healthcare by limiting direct out-of-pocket health expenditures (OOPHE). The study examines the effect of the NHIS on OOPHE and how it protects households against catastrophic health expenditures.

**Methods:**

Data was obtained from a cross-sectional representative household survey involving 2,430 households from three districts across Ghana. All OOPHE associated with treatment seeking for reported illness in the household in the last 4 weeks preceding the survey were analysed and compared between insured and uninsured persons. The incidence and intensity of catastrophic health expenditures (CHE) among households were measured by the catastrophic health payment method. The relative effect of NHIS on the incidence of CHE in the household was estimated by multiple logistic regression analysis.

**Results:**

About 36% of households reported at least one illness during the 4 weeks period. Insured patients had significantly lower direct OOPHE for out-patient and in-patient care compared to the uninsured. On financial protection, the incidence of CHE was lower among insured households (2.9%) compared to the partially insured (3.7%) and the uninsured (4.0%) at the 40% threshold. The incidence of CHE was however significantly lower among fully insured households (6.0%) which sought healthcare from NHIS accredited health facilities compared to the partially insured (10.1%) and the uninsured households (23.2%). The likelihood of a household incurring CHE was 4.2 times less likely for fully insured and 2.9 times less likely for partially insured households relative to being uninsured. The NHIS has however not completely eliminated OOPHE for the insured and their households.

**Conclusion:**

The NHIS has significant effect in reducing OOPHE and offers financial protection against CHE for insured individuals and their households though they still made some out-of-pocket payments. Efforts should aim at eliminating OOPHE for the insured if the objective for establishing the NHIS is to be achieved.

## Background

In 2000, the World Health Organisation (WHO) recognised fairness in health finance as one of its three core health system goals [1]. Fairness in health financing and protection against financial risk suggest that accessing healthcare should not impose untold hardship on affected households [2]. Promoting fairness through health system financing arrangements therefore has a wider social value as its implication goes beyond improving health status [3]. It is estimated that globally, about 150 million people face catastrophic healthcare expenditures (CHE) annually because of direct payments for healthcare while about 100 million are driven into poverty [4]. This is mainly because such people lack prepayment schemes and have to pay for healthcare at the point of service.

A well-functioning prepayment arrangement which facilitates effective risk-pooling and risk-sharing among the population including the poor has been identified as having a strong potential to improve financial protection against illness [4–6]. Some studies have however found partial or no impact of health insurance on out-of-pocket health expenditures (OOPHE) and catastrophic health expenditures depending on the structure and services offered by the scheme [7–11].

In Ghana, the introduction of user fees in the health sector in the 1980s and 1990s impeded access to health services, imposed a heavy financial burden on households and often led to worse health outcomes for majority of the population [12–15]. As a response to address these adverse effects, the National Health Insurance Scheme (NHIS) was introduced in 2004 following the passage of the National Health Insurance (NHI) Act (Act 650) in 2003 [16]. According to the NHI Act 650, Ghana was to establish a national health insurance policy that ‘ensures access to basic healthcare services to all residents’ [17]. The NHIS is therefore aimed at removing the financial barrier to health services by limiting OOPHE [18].

Membership in the NHIS is mandatory for all residents of the country including foreign nationals legally resident in Ghana [17]. To become a valid card holding member of the NHIS, it is necessary to actively register and pay a registration fee. Formal sector employees who contribute to the Social Security and National Insurance Trust (SSNIT), a mandatory 2.5% deduction is made on their social security contributions as their premium [16]. Adults (18–69 years) in the informal sector pay an annual premium of between 7.2 Ghana Cedis (US$4.8 in March, 2011) and Gh¢ 48.0 (US$32.0) depending on the district of registration [19]. Children under 18 years, pregnant women, elderly (≥70 years), SSNIT pensioners and indigents are exempted from paying the premium [17]. The NHIS has a comprehensive benefit package that covers about 95% of the country’s disease conditions [20]. The benefit package covers outpatient services, inpatient care and maternity care among others. The NHIS has an exclusion list of health problems which include cancer treatment other than breast and cervical cancers, HIV retroviral drugs, dialysis for chronic renal failure, among other tertiary services.

Formal health care services in Ghana are delivered through an integrated and multilevel health system comprising of a hierarchy of health facilities [21]. At the base are the Community Health Planning and Services (CHPS) zones followed by health centers at the sub-district level. These facilities provide mainly primary and community health services. At the next levels are the district and regional hospitals providing both primary and secondary services. At the apex are the tertiary hospitals which provide tertiary services [22]. There are over 4,069 health facilities in Ghana including CHPS, clinics, hospitals, maternity homes, pharmacies and laboratories. Health care services for NHIS members are provided by about 3,575 health facilities which have been accredited by the National Health Insurance Authority [23]. About 54% of the accredited health facilities are owned by government (i.e. publicly owned) while 39.8% are privately owned. Religious bodies owned 5.8% while quasi-governmental institutions owned the remaining 0.8% [23].

About 34% of Ghana’s population of 24.6 million are valid card holding members (i.e. active members) of the NHIS [19]. The NHIS has become the dominant source of internally generated funds (IGF) to health facilities, accounting for 79.4% of IGF in 2010 [24]. At the national level, the NHIS appears to be contributing to a decline in OOPHE. OOPHE as a percentage of total health expenditure has declined from 47% in 2000 to 30% in 2007 but went up to 37% in 2009 [21]. In 2011, the percentage was 29.1% and still far higher than the 15%-20% threshold recommended by the WHO [4]. This situation has been described as ‘appearing problematic’ because there is a strong correlation between the ratio of OOP health expenditures to total health expenditures and the incidence of catastrophic health expenditures among households [21, 22, 25].

At the household level, evidence of the effect of the NHIS on financial protection is scanty in Ghana though that is one of the core objectives of the NHIS [26, 27]. In an earlier study to assess the financial protection effect of the NHIS on households in Ghana, Nguyen et al. [26] reported that the NHIS significantly reduced the probability of catastrophic health expenditure by 0.5 to 1 percentage point depending on whether non-food expenditure or household income was used for the estimation. The study however observed that NHIS members made OOP payments even at NHIS accredited health facilities. In all, the insured made OOPHE which equalled 72% of what was incurred by the non-members. Since the study was conducted in the early stages of the implementation of the NHIS in 2007, some of the observed problems (e.g. shortages of drugs, payment for services and drugs that should be covered by insurance, payment for uncovered drugs and tests, etc.) were attributed to teething problems associated with the implementation [26, 28].

After ten years of implementation of the NHIS, it is imperative to assess the effect of the NHIS on household’s OOPHE in Ghana. A positive answer to the question on whether health insurance reduces OOPHE ‘would be essential to argue that health insurance can help prevent people from sliding into health-related poverty’ [6]. Against this background, we hypothesise that individuals insured with the NHIS would have significantly lower direct OOPHE when ill and their households are less likely to incur catastrophic health care expenditures compared to the uninsured.

## Methods

### Study design

This study was conducted in three selected districts across the three main ecological zones of Ghana. The districts were Kwaebibirem in the southern zone, Asutifi in the middle zone and Savelugu-Nanton in the northern zone. The data was cross-sectional and representative of households in the three districts based on the Ghana Statistical Service’s demarcation and mapping for each district. Twenty-seven (27) representative rural and urban census enumeration areas (EAs) were sampled and surveyed in each district. Assisted by the district statisticians with the EA maps, all households in each EA were listed. The complete list of households in each EA constituted the sampling frame from which 30 households were systematically sampled and interviewed. This resulted in a total of 810 households per district and 2,430 households in the three districts. The survey was conducted between February and April, 2011.

A household questionnaire was developed and administered to the household head by trained field enumerators. The household questionnaire had modules which covered household’s demographic profile, socioeconomic status, health insurance status of all members, general health of household members, household expenditures and household dwelling characteristics as well as assets ownership. The health module collected information on all reported illnesses and injuries which occurred in the household in the last 4 weeks preceding the survey. For households which reported an illness/injury, information was collected on the symptoms or type of illness/injury experienced and perceived severity of the illness/injury. Individuals who reported ill/injured were asked whether they sought health care and the source of treatment. All direct OOP expenditures associated with treatment seeking for the reported illness/injury were recorded. The direct OOP health expenditures included fees for consultation, diagnostic tests, medicines, medical supplies and all non-medical expenses including travel expenses, subsistence cost at the facility and all other related payments. Those who sought healthcare from outside the formal health system (self-medication, purchase of medicines from drug stores, traditional medicine, etc.) also reported all direct OOP expenditures made.

The household expenditure module gathered information on all expenditures made by the household during the past 4 weeks or 12 months depending on the nature of the expenditure item. The components included food and non-food expenditures including imputed values for home produced items such as food and housing as used in the Ghana Living Standard Surveys [29]. The health expenditures component of the non-food expenditures sub-group included all health and medical expenditures incurred during the period as well as expenditures on medical products, therapeutic appliances and equipment, health insurance premiums and fees as well as expenditures on preventive health such as the purchase of mosquito nets and repellents [29, 30]. All expenditures with recall periods of more than 4 weeks were adjusted to get estimates for the 4 week reference period.

Information was also collected on type of health facilities in the three districts. The Kwaebibirem district with a population of about 200,000 had one district hospital and two other non-governmental hospitals. There were three health centres, two clinics and 23 Community-based Health Planning and Services (CHPS) compounds. The Asutifi district with a population of 114,029 had 16 health facilities consisting of one mission hospital which serves as the district hospital, four public health centres, two clinics, four CHPS compounds and five private clinics. The Savelugu-Nanton district had one public district hospital, one private hospital, three health centres, five clinics and three CHPS compounds.

Ethical approval for the survey was obtained from the Institutional Review Board of the Noguchi Memorial Institute for Medical Research of the University of Ghana with a certified protocol number 069/11-12. Informed consent was also sought from all participating households.

### Statistical analyses

Statistical analyses for the paper were conducted on individual and household levels. At the individual level, the impact of the NHIS on OOPHE (direct cost of illness) was examined. The individual direct cost of illness was calculated as the sum of all the direct OOPHE on medical and nonmedical items related to the reported illness/injury [31, 32]. We compared the mean OOPHE by insured and uninsured sick persons. At the individual level, the analysis was based on the cost per illness episode from a single visit to the health facility.

At the household level financial protection was assessed by measuring the incidence and intensity of financial catastrophe due to OOPHE in the household for the reference period using the standard methods by O’Donnell et al. [33]. Household’s total out-of-pocket health expenditure (*T*) is defined as catastrophic if it exceeds a specified fraction (threshold) of total household expenditure (*x)* or total non-food expenditure [*x-f (x)*]*.* It is common to set this threshold (*z*) at 10% of total expenditure or 40% of total non-food expenditure [33–35]. We defined financial catastrophe in relation to total household non-food expenditure by using the 20% and 40% thresholds. The incidence of catastrophic health expenditure (CHE) which is referred to as the catastrophic payment headcount (*H*), measures the proportion of the sampled households whose OOPHE as a fraction of their total non-food expenditures exceeds the specified threshold (*z*). The catastrophic payment headcount (*H*) is estimated by

1 $$ H=\frac{1}{N}{\displaystyle {\sum}_{i=1}^N{E}_i}, $$

where *N* is the sample size and *E* is an indicator variable which equals 1 if a household *(i)* is classified as having incurred catastrophic health expenditure (*T*_*i*_*/[x*_*i*_*-f (x*_*i*_*)]* > *z*) and 0 if otherwise. The catastrophic payment headcount (*H*) however does not show the extent to which households incurring CHE exceeds the specified threshold. This is measured by the catastrophic payment overshoot (*O*) which shows the intensity of the catastrophic OOP health expenditure*.* The intensity therefore measures the average degree by which the OOP health expenditures as a fraction of total non-food expenditures exceed the specified threshold for all the sampled households. The overshoots (*O*) is defined as *O*_*i*_ *= E*_*i*_*((T*_*i*_*/ [x*_*i*_*-f (x*_*i*_*)]-z).* For the sampled households, the catastrophic payment overshoot (*O*) is estimated by

2 $$ O=\frac{1}{N}{\displaystyle {\sum}_{i=1}^N{O}_i}. $$

The incidence (*H*) and the intensity or overshoot (*O*) are related through the mean positive overshoot *(MPO = O/H).* The MPO measures the mean overshoot among households making CHE at a specified threshold in the sample. Results are compared among uninsured, partially insured and fully insured households to assess any significant effect of the NHIS. The household is defined as a person or a group of persons, who live together in the same dwelling, sharing the same house-keeping arrangements and are catered for as one unit [29]. The household was uninsured if none of its members was a member of the NHIS, partially insured if at least one member was insured or fully insured if all the members were insured at the time of the survey.

Finally, we explored the determinants of catastrophic health expenditure in the household by estimating binary logistic regression models. The purpose was to ascertain the independent effect of health insurance on the occurrence of CHE in the household by controlling for other confounding variables. The probability (*P*) of a household incurring CHE (*y*) is specified as: *P (y = 1|x) = exp (β’x) / 1 + exp (β’x),* where the dependent variable *y* = 1 if the household incurred CHE and *y* = 0 if the household did not incur CHE, *x* is a vector of the independent variables, *β is* the parameters [36]. The literature suggests that the probability of a household incurring CHE is a function of its socio-demographic characteristics such as the size and age composition, place of residence, health status of members, health insurance status of members, economic status (e.g. wealth status), type and place of treatment as well as the characteristics of the household head [37–41]. The household wealth status was generated by Principal Components Analysis (PCA) based on thirty-two items including households’ dwelling characteristics, access to utilities and ownership of consumer durables. Weights from the first principal component were used to generate the household wealth quintiles [42]. Two multiple logistic regression models were estimated. Model 1 was estimated for all households (2,418) while Model 2 was restricted to only users of NHIS accredited facilities for the recent reported illness (584). The model goodness-of-fit was assessed using Hosmer–Lemeshow test and the results showed that they were satisfactory (*p = 0.1936* for model1 and *p = 0.4711* for model 2) [43]. All statistical analyses were performed using Stata 12.

## Results

### Descriptive statistics

The results show that 9.8% (1,082) of the 11,089 household members in 36% of the surveyed households reported sick in the last four weeks preceding the survey. About 14.7% (633) of the insured household members compared to 6.6% (449) of the uninsured reported sick (Table 1). Nearly 50% of the uninsured sick were children compared to about 39% among the insured sick while there was a higher proportion of the elderly among the insured sick persons (*p < 0.01*). Fever/malaria was the commonest illness reported (39.0%). This was followed by gastrointestinal tract disorders (14.2 %) and musculoskeletal related complaints (11.6 %). The rest were disorders of the cardiovascular system (5.8%), skin disorders (5.1%) and injuries (3.7%) among others.

**Table 1:** Descriptive statistics of household members who reported sick by their health insurance status

Variable	Insurance status		Total (n = 1,082) %	N	Pearson’s $\boldsymbol{\upchi}$ ^2^
	Uninsured (n = 449) %	Insured (n = 633) %			
District					
Asutifi	35.2	44.9	40.9	442	26.038***
Kwaebibirem	34.5	37.8	36.4	394	
Savelugu-Nanton	30.3	17.4	22.7	246	
Age (years)					
< 18 yrs	49.4	38.9	43.2	468	25.729***
18-69	45.4	47.5	46.7	505	
≥ 70	5.1	13.6	10.1	109	
Sex					
Male	47.4	38.4	42.1	456	8.824***
Female	52.6	61.6	57.9	624	
Illness/symptoms					
Fever/malaria	40.6	37.9	39.0	422	28.651***
Gastrointestinal tract disorders	17.2	12.2	14.2	154	
Musculoskeletal related	9.8	13.0	11.7	126	
Injuries	3.4	3.9	3.7	40	
Respiratory system disorders	2.5	3.0	2.8	30	
Skin disorders	6.8	3.9	5.1	55	
Cardiovascular system disorders	2.5	8.1	5.9	63	
Eye infection	2.3	3.0	2.7	29	
Obstetric related complaints	0.9	1.9	1.5	16	
Other	14.1	13.3	13.6	147	
Perceived severity					
Very serious	24.4	29.1	27.2	292	4.422
Somewhat serious	50.2	50.0	50.1	538	
Not serious	25.3	20.9	22.7	244	
% of sick who sought care	93.8	95.3	94.6	1,024	1.160
Source of treatment (for those who sought any care)					
Regional/District hospital	19.5	39.3	31.4	317	117.423***
Public Health centre	17.2	28.4	24.0	242	
CHPS	2.8	3.0	2.9	29	
Private hospital/clinic	7.8	15.3	12.3	124	
Informal	52.8	14.0	29.4	296	
% that recovered after first visit	97.3	92.6	94.5	968	10.628***
% of inpatient (hospitalisation)	5.2	10.9	8.6	93	10.823***
% that received all prescribed medicines from NHIS accredited facilities	87.2	87.8	87.7	611	0.835
Mode of transport to place of treatment					
Foot	58.7	47.5	51.9	515	30.746***
Bicycle	4.4	2.5	3.2	32	
Motorcycle	5.1	5.0	5.0	50	
Car/bus	29.5	44.7	38.7	384	
Truck	2.3	0.3	1.1	11	

Source: Authors; household survey, 2011

*Significant at 10%; **Significant at 5%; ***Significant at 1%

Nearly 95% of the sick reported to have sought health care for their illnesses with no significant difference between the uninsured (93.8%) and the insured (95.3%). There was however a significant difference when it comes to the source of treatment chosen (*p < 0.01*). A significant proportion of the insured (39.3%) compared to 19.5% of the uninsured sought health care from the regional and district hospitals. More than half (52.7%) of the uninsured sought health care from informal sources including traditional sources, self treatment and purchase of unprescribed medicines from drugstores.

Only 8.6% of the sick were hospitalised with a higher proportion among the insured (10.9%) than the uninsured (5.2%) (*p < 0.01*). A similar proportion (87%) of uninsured and insured patients received all their prescribed medicines from the NHIS accredited facilities visited. Majority (51.9 %) of the sick visited the place of treatment on foot while 40% went by a car or bus. The insured were more likely (44.7%) to travel by car or bus to seek health care than the uninsured (29.5%) (*p < 0.01*).

### Direct cost of illness for treatment sought by health insurance status

Table 2 presents the mean direct cost of illness for treatment sought for insured and uninsured sick persons. The total direct cost of out-patient care including both medical and nonmedical costs for the recent illness was significantly higher among the uninsured (Gh¢25.50/US$17.00) compared to the Gh¢6.65 (US$4.43) for insured patients (*p < 0.01*). The direct medical cost of out-patient care incurred by the uninsured was 10.8 times (Gh¢21.30/US$14.20) higher than that of the insured (Gh¢1.97/US$1.31). The insured however recorded a higher cost for the cost of prescribed drugs purchased from outside the health facility visited though the difference was not statistically significant (Gh¢14.27/US$9.51 vs. Gh¢10.62/US$7.08, *p > 0.1*). Though transport cost for seeking out-patient care was generally low (Gh¢1.57/US$1.05), it was significantly higher for the insured compared to the uninsured (Gh¢2.11/US$1.41 vs. Gh¢1.28/US$0.85, *p < 0.01*). It is however important to note that transport costs are not covered by the NHIS.

**Table 2:** Mean direct cost of illness by type of care sought and health insurance status (in Ghana cedis^a^)

Variable	Insurance status			*T-stat* ^*b*^	Frequency (n)		
	All	Uninsured	Insured		All	Uninsured	Insured
Out-patient services cost							
Total direct cost of illness (medical & nonmedical)	11.67 (18.85)	25.50 (38.21)	6.65 (15.61)	8.661***	619	167	452
Total direct medical cost at NHIS facility (excluding non-medical costs)	7.19 (24.69)	21.30 (42.88)	1.97 (7.60)	9.209***	619	167	452
Cost of prescribed drugs bought from outside facility	13.16 (19.67)	10.62 (9.21)	14.27 (22.80)	−0.636	56	17	39
Transport cost	1.57 (4.73)	1.28 (4.37)	2.11 (4.95)	2.641***	619	167	452
Inpatient services cost							
Total direct cost of illness (medical & nonmedical)	66.62 (66.26)	86.73 (87.48)	44.25 (53.22)	2.478***	71	19	52
Total direct medical cost at facility (excluding nonmedical costs)	23.63 (41.12)	54.64 (59.73)	12.20 (24.07)	3.686***	52	14	38
Transport cost	6.47 (8.52)	5.39 (4.95)	6.93 (9.69)	−0.582	50	15	35
Informal care cost^c^							
Total direct cost of illness	4.79 (7.20)	5.58 (5.77)	6.41 (7.97)	−0.738	280	196	84

Authors; computed from household survey data, 2011

Notes: Figures in parentheses are 95% confidence intervals

^a^In Ghana cedis. US$1 = 1.5003 in March, 2011

^b^*Significant at 10% (*p < 0.1*); **Significant at 5% (*p < 0.05*); ***Significant at 1% (*p < 0.01*)

^c^Informal sources of treatment included traditional care, self treatment and purchase of unprescribed drugs from chemical shops

For the few respondents who sought in-patient care (8.6%), the uninsured incurred a significantly higher total direct cost (Gh¢86.73/US$57.81) than the insured (Gh¢44.25/US$29.49) (*p < 0.01*). Finally, there was no significant difference in the total direct cost for those who sought care from informal sources though the insured paid a little higher (Gh¢6.41/US$4.27) than the uninsured (Gh¢5.58/US$.72).

### Incidence and intensity of catastrophic health expenditures at the household level

The results show that 46.2% of the households interviewed were uninsured. About 26% were partially insured while 28.2% were fully insured. The households’ mean monthly non-food expenditure was significantly higher for fully insured households (Gh¢111.74/US$74.48) compared to the uninsured households (Gh¢97.77/US$65.17) (*p < 0.01*). The partially insured households recorded the highest mean OOPHE (Gh¢13.02/US$8.68) during the reference period (Table 3). About 44% of the partially insured and fully insured households reported at least one acute illness in the last four weeks preceding the survey compared to only 25.5% of the uninsured households (*p < 0.01*). A significant proportion of the fully and partially insured households reported to have at least a member with a chronic illness (*p < 0.01*).

**Table 3:** Summary of household non-food expenditure, OOPHE and health conditions

Variable	Health insurance status			Total (n = 2,418)	F-Test^b^
	Uninsured (n = 1,117)	Partially insured (n = 620)	Fully insured (n = 681)		
Mean household non-food expenditure (Gh¢)^a^	97.77 (76.15)	117.71 (86.30)	129.21 (100.26)	111.74 (87.17)	30.18***
Mean per capita household non-food expenditure (Gh¢)	32.65 (34.02)	25.87 (23.06)	48.57 (58.08)	35.39 (44.89)	47.11***
Mean OOP health expenditure (Gh¢)	7.66 (21.31)	13.02 (34.05)	9.62 (19.79)	9.59 (24.93)	9.29***
Mean OOP health expenditure as % of non-food expenditure (%)	7.49 (13.09)	9.33 (12.86)	8.77 (11.30)	8.32 (12.57)	4.85***
Households with illness in the last 4 weeks (%)	25.52 (43.61)	44.52 (49.73)	44.93 (49.78)	35.86 (47.97)	50.22***
No. of illness per household in the last 4 weeks (mean)	1.20 (0.55)	1.33 (0.69)	1.21 (0.51)	1.25 (0.59)	4.39**
Having a member with chronic illness (%)	7.61 (26.52)	16.45 (37.10)	19.53 (39.67)	13.23 (33.89)	30.66***

Source: Authors; computed from household survey data, 2011

Notes: Figures in parentheses are the standard deviations of the means

^a^In Ghana Cedis. US$1 = 1.5003 in March 2011

^b^F-test for equal means. *Significant at 10% (*p < 0.1*); **Significant at 5% (*p < 0.05*); ***Significant at 1% (*p < 0.01*)

Table 4 presents the incidence (headcount) and intensity (overshoot) of catastrophic health expenditures (CHEs) among the surveyed households. For all households in the sample, 11.4% (275) of them incurred CHEs at the 20% threshold with no significant difference in terms of their insurance status (*p = 0.444*). The MPO shows that the uninsured households which made CHEs at the 20% threshold on the average spent 39.4% (20% + 19.45%) of their total monthly non-food expenditure on health care compared to 36.44% (20% + 14.44%) among the partially insured and 34.9% (20% + 14.86%) among the fully insured households. At the 40% threshold, 3.6% (88) of the households made CHE (i.e. Uninsured = 3.7%, Partially and Fully insured = 2.9%). For these households incurring CHE at the 40%, the fully insured households exceeded the threshold by 14.3% compared to 16.7% for the uninsured and 20.5% for the partially insured though the difference was not statistically significant (*p = 0.323*).

**Table 4:** Incidence and intensity of catastrophic OOP health expenditures relative to non-food expenditure

Threshold level, z										
Indicator	20% for all households					40% for all households				
	Uninsured (n = 1117)	Partially insured (n = 620)	Fully insured (n = 681)	All (n = 2418)	*P-value* ^*a*^	Uninsured (n = 1117)	Partially insured (n = 620)	Fully insured (n = 681)	All (n = 2418)	*P-value* ^*a*^
	20%					40%				
Head count (%)	10.74	12.74	11.16	11.37	0.444	4.03	3.71	2.94	3.64	0.485
Standard Error (%)	0.93	1.34	1,27	0.64		0.59	0.76	0.65	0.38	
Overshoot (%)	2.09	2.09	1.66	1.97	0.467	0.07	0.08	0.04	0.06	0.288
Standard Error (%)	0.24	0.33	0.26	0.16		0.13	0.19	0.13	0.08	
Mean Positive Overshoot (%)	19.45	16.44	14.86	17.32	0.135	16.69	20.54	14.29	17.16	0.323
Restricted to users of NHIS accredited health facilities for the recent illness/injury										
Indicator	20%					40%				
	Uninsured (n = 125)	Partially insured (n = 208)	Fully insured (n = 251)	All (n = 584)	*P-value* ^*a*^	Uninsured (n = 125)	Partially insured (n = 208)	Fully insured (n = 251)	All (n = 584)	*P-value* ^*a*^
Head count (%)	56.80	25.96	17.53	28.94	0.0000	23.20	10.10	5.98	11.13	0.0000
Standard Error (%)	4.45	3.05	2.40	1.88		3.79	2.09	1.50	1,30	
Overshoot (%)	12.35	5.11	3.09	5.79	0.0000	4.23	1.89	0.09	1.98	0.0001
Standard Error (%)	1.49	0.86	0.61	0.53		0.90	0.48	0.33	0.30	
Mean Positive Overshoot (%)	21.74	19.68	17.64	20.01	0.4530	18.27	18.68	15.66	17.80	0.7900

Source: Authors; computed from household survey data, 2011

*P*-values based on F-test for equal means

Restricting the analysis to only the users of NHIS accredited health facilities for the recent illness/injury revealed the significant effect of the NHIS on OOP health expenditures. At the 40% threshold, 11.1% (65) of the households made CHE but it was as high as 23.2% (29) among the uninsured compared to only about 6.0%% (15) among the fully insured households (*p < 0.001*). The fully insured household also had the lowest MPO of 15.7% though the difference was not significant (*p = 0.790*).

### Determinants of catastrophic health expenditure at the household level

The multiple logistic regression estimates for all the surveyed households (Model 1) showed that the number of children under five years in the household, household size, health status of household members, sex of the household head, longer distance to the nearest health facility and more importantly the health insurance status of the household were all significant determinants of CHE by households (Table 5). For instance, households with at least one member having a chronic illness were 94% higher than those without a chronic illness in incurring CHE. Households which experienced hospitalisation of a member were over 7.0 times more likely than those without hospitalisation to incur CHE in both models.

**Table 5:** Logistic regression estimates of the determinants of catastrophic health expenditure (CHE ≥ 40% of non-food expenditure)

Variable	Model 1			Model 2		
	All households			Users of NHIS accredited facilities		
	Odds Ratio	95% CI for OR		Odds Ratio	95% CI for OR	
		Lower	Upper		Lower	Upper
Personal characteristic						
No. of under 5 years in household	1.49**	1.07	2.08	1.20	0.80	1.82
No. of ≥70 years in household	1.16	0.62	2.16	1.33	0.63	2.81
Household size (# of persons)	0.90*	0.79	1.02	0.91	0.78	1.07
Health status						
Illness per capita (last 4 weeks)	1.24*	0.96	1.60	1.23	0.90	1.69
Having a member with chronic illness (None = 0)	1.94*	0.93	4.03	1.77	0.75	4.20
A member sought heath care from a formal health facility-last 4 weeks	8.49***	4.70	15.32			
A member hospitalised-last 4 weeks	7.08***	3.63	13.83	7.41***	3.62	15.17
Health insurance status (Uninsured =0)						
Partially insured household	0.35***	0.18	0.65	0.36***	0.17	0.73
Fully insured household	0.24***	0.12	0.49	0.18***	0.08	0.42
Household/Head characteristics						
Residence: Urban (Rural = 0)	0.89	0.46	1.73	0.76	0.34	1.72
Female-headed (male = 0)	1.82*	0.96	3.45	2.25**	1.04	4.87
Marital status of head (never married = 0)						
Married/in-union	2.10	0.46	9.65	0.83	0.15	4.47
Divorced/widowed	1.27	0.25	6.42	0.27	0.04	1.76
Education of head (No formal educ = 0)						
Primary	0.26**	0.10	0.68	0.40*	0.14	1.15
Junior secondary/middle school	0.51**	0.27	0.96	0.44**	0.20	0.98
Senior secondary or higher	0.77	0.28	2.16	0.47	0.12	1.81
Sector of employment of head (Unemployed = 0)						
Formal	1.39	0.36	5.34	1.78	0.32	9.85
Informal	1.35	0.52	3.52	2.01	0.59	6.84
Wealth quintile (First = 0)						
Second	0.62	0.32	1.20	0.60	0.26	1.39
Third	0.68	0.33	1.41	0.84	0.35	2.06
Fourth	0.53	0.23	1.22	0.51	0.18	1.48
Fifth	0.45*	0.17	1.15	0.71	0.23	2.19
Health system/community factors						
Distance to nearest health facility (Ref = <2km)						
Between 2-5 km	1.29	0.63	2.61	1.43	0.61	3.32
More than 5 km	1.99*	0.88	4.50	2.06	0.73	5.81
District (Savelugu-Nanton = 0)						
Asutifi	1.64	0.78	3.46	1.03	0.41	2.56
Kwaebibirem	1.92	0.88	4.22	1.04	0.39	2.77
No. of observations	2,418			584		
Pseudo R^2^	0.2661			0.2004		
LR test $\boldsymbol{\upchi}$ ^2^ (df), *p-value*	201.13 (26)	<0.001		81.75 (25)	<0.001	
H-L goodness-of-fit test (8) $\boldsymbol{\upchi}$ ^2^; p-value	11.15	0.1936		7.62	0.4711	

*Significant at 10% (*p < 0.1*); **Significant at 5% (*p < 0.05*); ***Significant at 1% (*p < 0.01*)

The results from the two models showed that membership in the NHIS significantly reduced the probability of a household experiencing CHE. Partially insured households were 2.9 times less likely to experience CHE while fully insured households were 4.2 times less likely after controlling for other variables (Model 1). Restricting the analysis to only households which sought health care from NHIS accredited health facilities for the recent reported illness (Model 2) revealed that fully insured households were 5.5 times less likely to incur CHE while the partially insured were 2.8% less likely. Finally, households living more than five kilometres from the nearest NHIS accredited health facility were 1.9 times more likely to incur CHE relative to those within less than two kilometres.

## Discussion

This study examines the extent to which Ghana’s National Health Insurance Scheme (NHIS) protects its members against the financial consequences of ill-health. The results show that the insured persons were more likely to seek formal health care when ill and also have significantly lower direct OOPHE because of the NHIS. Though the insured patients were more likely to travel to the health facilities by car and therefore incurred relatively higher travel expenses, they still had lower mean OOPHE. This could suggest that with the elimination or reduction in OOPHE at NHIS accredited health facilities, the insured patients could afford to travel to seek appropriate health care when sick. In a recent study in the Kassena-Nankana district of the Upper East region of Ghana, Dalaba et al. [44] observed that insured patients with the NHIS were more likely to seek formal health care when sick with malaria.

The WHO [4] has observed that elimination of direct OOP payments at health facilities does not necessarily remove all the financial barriers to access. This is because nonmedical expenditures such as travel and subsistence expenses as well as informal payments at the health facilities could still impede access. We observed that travelling longer distances to seek health care could contribute to high OOPHE due to transport cost. While transport cost is beyond the benefit package of the NHIS, any improvement in reducing the physical barrier to health care could help reduce the direct cost of illness.

It has been observed that the ability for a particular health insurance programme to protect its members against the financial consequences of ill-health, to a large extent, depends on its design, the benefit package and purchasing arrangements with service providers [6]. This may explain the lack of consistency on whether health insurance protects against catastrophic health expenditures [6, 11]. In the case of the NHIS, the benefit package covers about 95% of disease conditions in Ghana yet some insured patients still made OOP payments even at NHIS accredited health facilities. One is however not certain whether these payments were for services or drugs not covered under the NHIS or they were unauthorised co-payments. Though this study did not have explicit information on informal payments at health facilities, there is anecdotal evidence of informal payments by insured patients who are sometimes made to make OOP payments for unapproved prescribed medicines at NHIS accredited health facilities [22, 24]. While the insurance authority has recently established a call centre for aggrieved clients to call to complain about any unauthorised co-payments at accredited health facilities, its effectiveness is yet to be seen. On the issue of medical supplies, about 12% of the insured and uninsured patients did not get all their prescribed medicines from the facilities they visited. The fact that insured patients paid higher than the uninsured (Gh¢14.27/US$9.51 vs. Gh¢10.62/US$7.08) for drugs purchased from outside the facility raises a major concern which requires further investigations. This is because we do not have any explanation for this observation. However, ensuring that NHIS accredited health facilities are stocked with essential medicines and medical supplies could help reduce the direct medical cost associated with the purchase of drugs from outside accredited health facilities.

The treatment seeking behaviour of a patient can also affect the direct cost of illness. Our results show that seeking health care from informal sources could be expensive. Insured patients who sought health care from informal sources spent Gh¢6.41/US$4.27 which was closer to the total direct cost of Gh¢6.65/US$4.43 for seeking formal health care. This is because informal facilities providing traditional and alternative medicines are not covered by the NHIS. As the NHIS seeks to expand its coverage, it can consider granting accreditation to alternative medicine practitioners who have been approved to practice by the Ministry of Health in view of the growing importance of that sub-sector in Ghana’s health delivery system [45]. The reported high use of informal treatment among the uninsured patients equally deserves attention perhaps through more public education. A similar observation is reported where 33% of the uninsured patients went directly to the drugstore to buy medicines when ill without prescription compared to only 7% among the insured in Ghana [21]. As emphasised by van Doorslear et al. [31] impoverishment due to health payments is ‘more disturbing when it arises from spending on self-prescribed medicines that have little or no positive effect’.

At the household level, it was observed that about 3.6% of the households incurred CHE at the 40% threshold. This was comparable to results from similar studies in Tunisia (4.54%), India (3.44%), Vietnam (5.97%), Nepal (4.56%) and Kenya (4.6%) [39, 46]. Our results did not show any significant difference in the incidence of CHE at the 40% threshold for all the households surveyed. This observation of no significant difference could be attributed to the fact that many of the uninsured patients purchased drugs from drugstores where the cost could be lower for unprescribed drugs. It is known that households with low incomes and without health insurance could be deterred from using formal health care services because of the high OOP payments associated with it. Such households could even forgo treatment or resort to self-treatment and incur lower OOPHE [4, 35, 47]. For instance, it has been reported that the uninsured in the poorest wealth quintile in Ghana are more likely to forgo treatment when ill [48]. Focusing on households which used NHIS accredited facilities clearly reveals the significant financial protection effect of the NHIS. The fully and partially insured households experienced significantly lower incidence and intensity of CHE. The positive and significant protective effect of the NHIS was also confirmed by our results from the regression analysis of the determinants of CHE. This is consistent with the earlier findings on the subject matter in Ghana by Nguyen et al. [26].

Notwithstanding the positive effect of the NHIS, the results show that about 6.0% of fully insured and 10.0% of the partially insured households experienced CHE though they used NHIS accredited health facilities. This is worrying because those households on the average spent more than 55% of their non-food expenditures on health payments as shown by the high mean positive overshoots (MPOs). It has been argued that OOPHE by households are involuntary and depending on the magnitude, such expenditures could result in cuts in consumption of basic necessities which undermines household’s welfare and could lead to asset sales, running down of savings, indebtedness and impoverishment [5, 31, 32, 35, 49–51]. The high MPOs for the partially insured and the fully insured households could perhaps be attributed to the fact that they reported more illnesses and had a higher proportion of members with chronic illnesses. The fact that these factors are significant determinants of the incidence of CHE suggests that the NHIS should re-examine the benefit package for chronic diseases which are currently not covered.

While this study has provided some highlights on the financial protection effects of the NHIS, it has some limitations. First of all, OOPHE at the individual level did not include cost of preventive health seeking though it was captured in estimating households total OOPHE. The study did not explore how households which made catastrophic health payments coped with the situation and its impoverishment effects, if any. We should also be mindful that the expenditure data used for the various measurements were self-reported by households and could suffer from recall errors though quality control measures were adhered to during the data collection exercise. Secondly, variations in the seasonality of diseases and income levels in the study districts which could affect the level OOPHE were not considered. The number of reported illnesses especially malaria could have been higher if data had been collected during the raining season when incomes are also expected to be high because that is the main agricultural season. But this was not expected to impact differently on both the insured and uninsured. Informal payments at health facilities were not specifically captured because what constituted informal payment was difficult to ascertained during the pre-testing of the questionnaire. It was assumed to be part of the ‘other’ cost category. The study also could not assess the interactive effects of wealth and health insurance in the estimation of the determinants of CHE because it presented serious multicollinearity problems.

## Conclusion

From our results, we can conclude that though the NHIS had not completely eliminated catastrophic health expenditures among its members, it provides a significant financial protection in times of illness for insured households. This is consistent with the general observation that the NHIS is making positive impacts on reducing the financial barriers to health care in Ghana [21, 24, 25]. It is possible to suggest that the ‘savings’ from the lower direct cost of health services for the insured could be used for transport to obtain ‘better’ care at formal health care providers to better their health. It is believed that improvement in the physical access to health services, supply of medical supplies and medicines at accredited health facilities and ensuring that accredited health institutions adhere strictly to the NHIS benefit package could protect insured households against the financial consequences of health shocks.

## Abbreviations

CHE: Catastrophic Health Expenditure
CHPS: Community-based Health Planning and Services
DMHIS: District Mutual Health Insurance Schemes
IRB: Institutional Review Board
LMIC: Low-and Middle-Income Countries
MPO: Mean Positive Overshoot
NHI: National Health Insurance
NHIA: National Health Insurance Authority
NHIS: National Health Insurance Scheme
NMIMR: Noguchi Memorial Institute for Medical Research
OOP: Out-of-Pocket
OOPHE: Out-of-Pocket Health Expenditures
SSNIT: Social Security and National Insurance Trust
WHO: World Health Organisation
